# Overexpression of KLF4 promotes cell senescence through microRNA-203-survivin-p21 pathway

**DOI:** 10.18632/oncotarget.11200

**Published:** 2016-08-11

**Authors:** Qing Xu, Mei Liu, Ju Zhang, Liyan Xue, Guo Zhang, Chenfei Hu, Zaozao Wang, Shun He, Lechuang Chen, Kai Ma, Xianghe Liu, Yahui Zhao, Ning Lv, Shufang Liang, Hongxia Zhu, Ningzhi Xu

**Affiliations:** ^1^ Laboratory of Cell and Molecular Biology and State Key Laboratory of Molecular Oncology, Cancer Hospital, Chinese Academy of Medical Sciences and Peking Union Medical College, Beijing, China; ^2^ Division of Proteomics, Beijing Institute of Genomics, Chinese Academy of Science, Beijing, China; ^3^ Department of Pathology, Cancer Hospital, Chinese Academy of Medical Sciences and Peking Union Medical College, Beijing, China; ^4^ State Key Laboratory of Biotherapy and Cancer Center/Collaborative Innovation Center for Biotherapy, West China Hospital, Sichuan University, Chengdu, P. R. China

**Keywords:** KLF4, p21, survivin, miR-203, senescence

## Abstract

Krüppel-like factor 4 (KLF4) is a transcription factor and functions as a tumor suppressor or tumor promoter in different cancer types. KLF4 regulates many gene expression, thus affects the process of cell proliferation, differentiation, and apoptosis. Recently, KLF4 was reported to induce senescence during the generation of induced pluripotent stem (iPS) cells, but the exact mechanism is still unclear. In this study, we constructed two doxycycline-inducing KLF4 cell models, and demonstrated overexpression of KLF4 could promote cell senescence, detected by senescence-associated β-galactosidase activity assay. Then we confirmed that p21, a key effector of senescence, was directly induced by KLF4. KLF4 could also inhibit survivin, which could indirectly induce p21. By miRNA microarray, we found a series of miRNAs regulated by KLF4 and involved in senescence. We demonstrated that KLF4 could upregulate miR-203, and miR-203 contributed to senescence through miR-203-survivin-p21 pathway. Our results suggest that KLF4 could promote cell senescence through a complex network: miR-203, survivin, and p21, which were all regulated by overexpression of KLF4 and contributed to cell senescence.

## INTRODUCTION

Krüppel-like factor 4 (KLF4/GKLF/EZF) is a zinc finger transcription factor that belongs to KLF transcription factor family. KLF4 functions as a context-dependent oncogene and tumor suppressor gene [1, 2]. KLF4 was reported downregulated in esophageal cancer, gastric cancer, colon cancer, kidney cancer, liver cancer and bladder cancer [3–8], but overexpressed in breast cancer [9, 10]. Moreover, clinical evidences indicated that inactivation of KLF4 was also associated with cancer progression and poor prognosis in some type of cancers [11, 12].

As a transcriptional factor, KLF4 can activate or repress gene expression through directly binding on promoter regions. For example, KLF4 could activate expression of p21 (also known as CIP1 and WAF1) [13], while repress the expression of Cyclin E [14]. KLF4 is involved in regulation of cell proliferation, differentiation and apoptosis [15–17]. Overexpression of KLF4 inhibits cell proliferation in cell lines and suppresses carcinogenesis and tumor metastasis in mice model [6]. On the contrary, deletion of KLF4 in mice model promotes carcinogenesis in many types of cancers [18, 19].

During the process of induced pluripotent stem cells, overexpression of KLF4 could induce cell senescence in fibroblast cells. And the reprogramming-induced senescence (RIS) was thought to be an important factor and a huge barrier, which resulted in low efficiency for generation of iPS cells [20–22]. On the other hand, Liu, et.al reported that in KLF4 gene knockout mice model, Klf4^−/−^ MEFs (mouse embryonic fibroblasts) could enter senescence earlier than Klf4^−/−^ MEFs [23].

Cellular senescence refers to the state of stable, irreversible growth arrest of a cell, always through p53/p21 and/or p16/Rb pathways [24]. Cell senescence has been regarded as a barrier of carcinogenesis *in vivo*, acting as an important tumor suppressor. However, recent studies revealed that although cell senescence initially functioned as a tumor suppressive factor, it might eventually exhibit some tumor-promoting effects [25].

Although oncogene-induced senescence has been intensively investigated for many years, but, whether and how tumor suppressor gene, such as KLF4, induces cell senescence is not fully explored yet. Here, we try to investigate KLF4 induced senescence in cancer cells and further decipher the molecular mechanisms of the KLF4-induced senescence.

## RESULTS

### KLF4 overexpression induces cellular senescence *in vitro*

**Figure 1:**
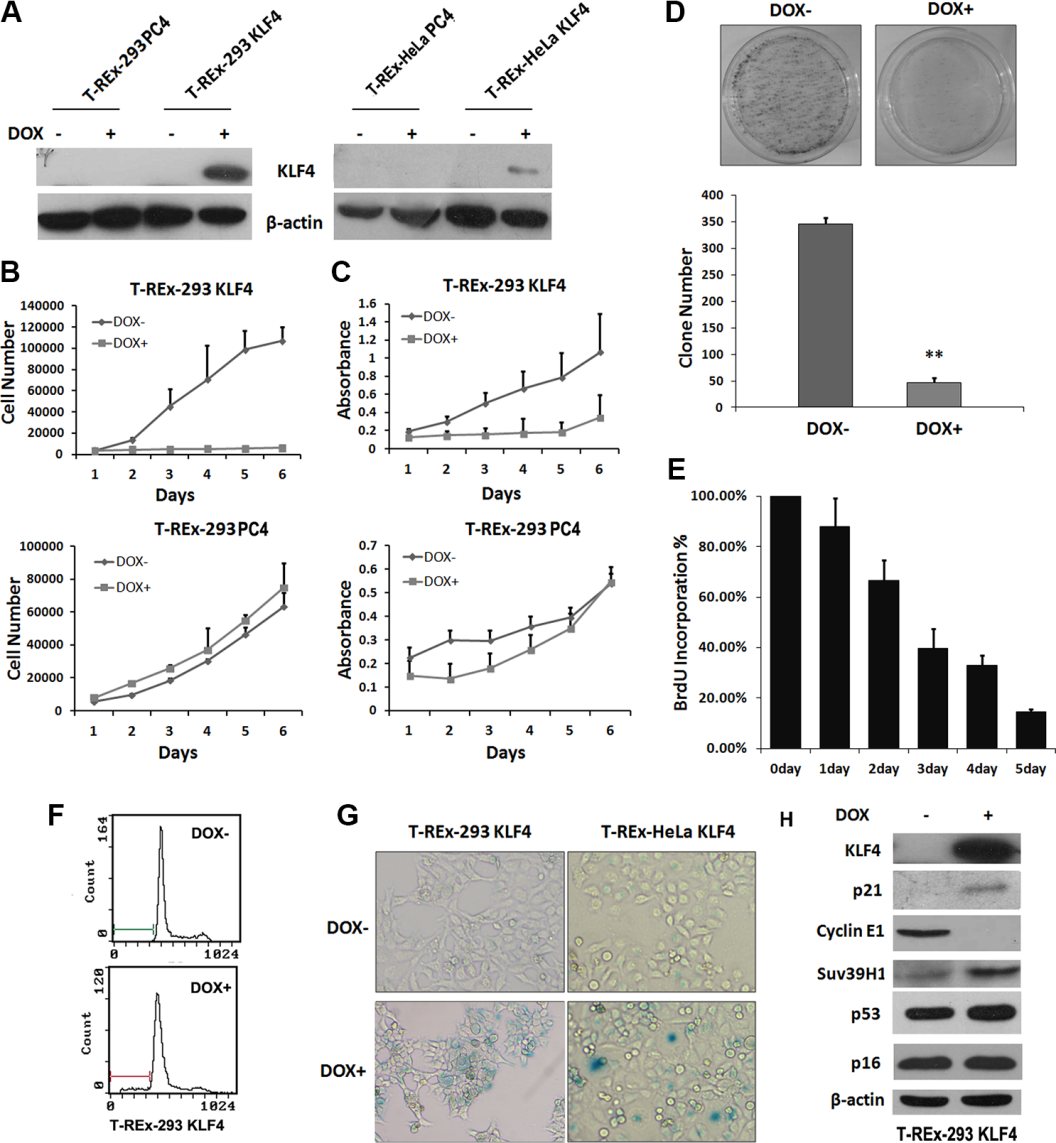
Overexpression of KLF4 induced cellular senescence in epithelial cells (**A**) KLF4 expression in KLF4 inducible expression system. KLF4 inducible cell lines were treated with 2 μg/ml DOX for 24 hours, and KLF4 expression was detected by Western blotting. (**B**) Growth curves and (**C**) MTT assay of T-REx-293 KLF4 and T-REx-293 PC4 cells after DOX treatment for o to 6 days. Results represent means ± s.d (*n* = 3). (**D**) Colony formation assay of T-REx-293 KLF4 cells. Representative clone formation photos were presented and colony number was counted. Bars represent the mean ± SD (*n* = 3). **p < 0.01. (**E**) BrdU incorporation assay of T-REx-293 KLF4 cells. (**F**) Flow cytometry assay of T-REx-293 KLF4 cells with or without DOX for 72hrs. (**G**) Detection of senescence in KLF4 inducible cells. T-REx-293 KLF4 and T-REx-HeLa KLF4 cells were seeded into 6-well plates, three days after DOX treatment, cellular senescence was detected by SA-β-Gal staining assay. Bars represent the mean ± SD of three independent experiments. (**H**) Western blotting analysis of senescence related protein in T-REx-293 KLF4 cells with or without DOX treatment for 3 days.

To investigate the role of KLF4 in cell senescence, we constructed two stable cell lines, T-REx-293 KLF4 and T-REx-HeLa KLF4. As shown in Figure 1A, KLF4 expression could be induced by 2 μg/ml doxycycline in both cell lines. KLF4 overexpression significantly suppressed cell growth (Figure 1B and 1C). In T-REx-293 KLF4 cells, KLF4 overexpression could suppress the colony formation (Figure 1D). DNA synthesis, measured by BrdU incorporation assay, was inhibited about 84 percent by induction of KLF4 for 5 days (Figure 1E). The percentage of apoptosis cells only increased from 0.93% to about 9.81% (Figure 1F). Then we characterized senescence in KLF4 overexpressed cells. Senescence-associated β-galactosidase activity (SA-β-gal) markedly increased in cells with KLF4 overexpression (Figure 1G). Transient overexpression of KLF4 could also induce cellular senescence in T-REx-293, AGS, and SW480 cells (Figure S1). We also detected the senescence related proteins by Western Blot, and found that p21^WAF1/CIP1^ protein was strongly accumulated after KLF4 overexpression, accompanied by reduced expression of Cyclin E1 and increased expression of Suv39H1 [26], but no significant variation of p53 and p16^Ink4a^ (Figure 1H). Our data suggest that KLF4 could induce cell senescence. K409Q mutation of KLF4 might not affect the function of KLF4 in inducing senescence. (Figure S2).

### KLF4 induces senescence though directly regulating p21 transcription

**Figure 2:**
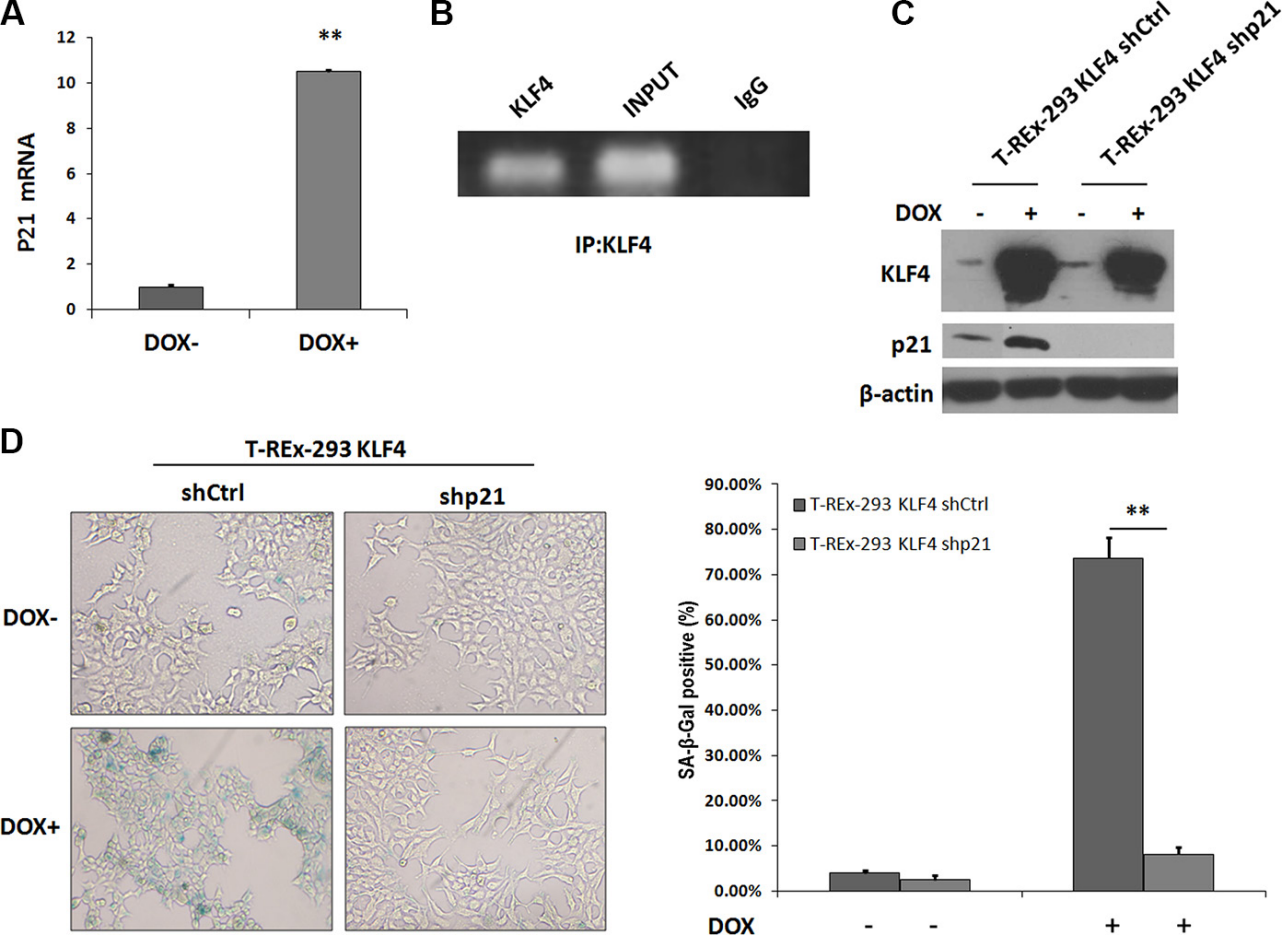
p21 expression increased in KLF4-induced senescence (**A**) p21 mRNA detected by Real-time PCR. T-REx-293 KLF4 cells were treated with DOX for 72 h and harvested for RNA extraction. Bars represent the mean ± SD (*n* = 3). **p < 0.01. (B) PCR result of KLF4 binding site of p21 gene promoter pulled down by ChIP. T-REx-293 KLF4 treated with DOX for 72 h were harvested and subjected to immunoprecipitation with either anti-KLF4 antibody or mouse IgG. Input DNA was applied as a positive control, and immunoprecipitation of IgG as a negative control. (**C**) p21 expression detected by Western blotting. T-REx-293 cells were transfected with shp21 and shCtrl plasmids and harvested after 72 h. (**D**) Representative SA-β-gal staining photos(magnification 100×) and percentage of senescence cells. Bars represent the mean ± SD (*n* = 3). **p < 0.01.

KLF4 has been reported to activate p21(WAF1/Cip1) through a specific Sp1-like cis-element in p21(WAF1/Cip1) proximal promoter [13]. We found that p21 mRNA level was induced by KLF4 overexpression (Figure 2A), and KLF4 could bind to the promoter region of p21 gene, confirmed by ChIP assay (Figure 2B). We further transfected p21 siRNA plasmids (shp21) into T-REx-293 KLF4 cells. When p21 protein was knocked down (Figure 2C), KLF4 induction could induce only about 8 percent of senescent cells, comparing with more than 70% senescent cells in control cells (Figure 2D). Our results suggest that increased expression of p21, directly regulated by KLF4, is essential to KLF4 induced senescence.

### KLF4 induces senescence though survivin-p21 pathway

**Figure 3:**
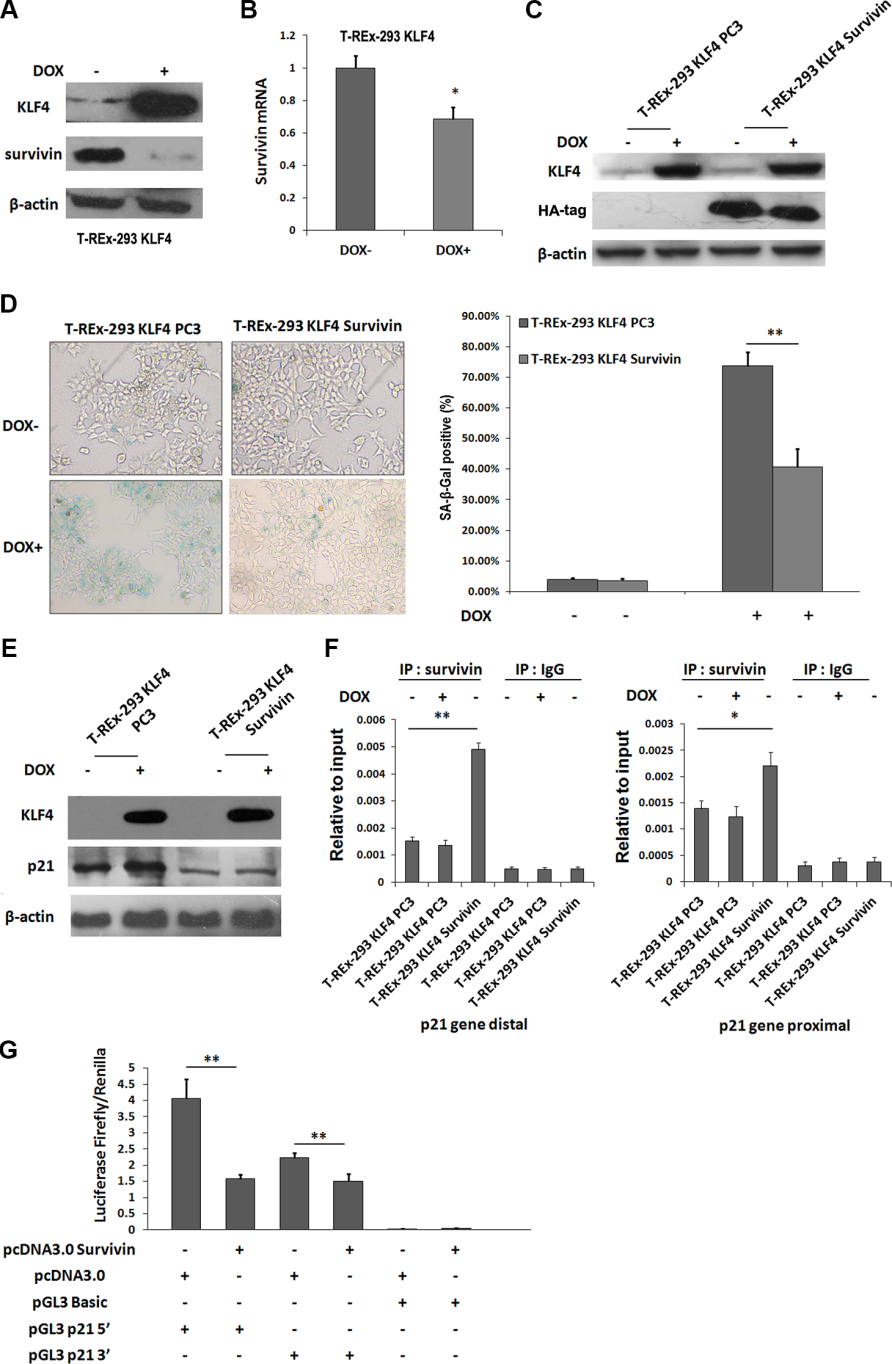
Survivin was involved in KLF4-induced senescence (**A**) Expression of survivin protein and (**B**) mRNA with or without DOX treatment of T-REx-293 KLF4 cells. Bars represent the mean ± SD (*n* = 3). *p < 0.05.(**C**) Expression of exogenous survivin detected by Western blotting. (**D**) Representative SA-β-gal staining photos and percentage of senescence cells. Bars represent the mean ± SD (*n* = 3). **p < 0.01. (**E**) Western blotting analysis of p21 in T-REx-293 KLF4 Survivin and T-REx-293 KLF4 PC3 cells. (**F**) Quantitative PCR analysis of direct binding of survivin to p53 distal (left) or proximal (right) binding site of p21 gene promoter. Anti-survivin antibodies were used for immunoprecipitation. Input DNA was used as internal control for quantification, and immunoprecipitation of IgG as negative control. Bars represent the mean ± SD of two independent experiments. *p < 0.05, **p < 0.01. (**G**) Reporter assay of p21 promoter region. T-REx-293 cells were transfected with pcDNA3.0 HA-Survivin or pcDNA 3.0 plasmids, pGL3 Basic, pGL3 p21 plasmids, and pRL-TK plasmids and assayed for p21 luciferase activities. Firefly/Renilla luciferase ratios were used to calculate fold induction. All experiments were performed in triplicated. **p < 0.01.

Our previous study showed survivin could be directly downregulated by KLF4 [27]. In camptothecin treated H1299 cells, Survivin expression is sustained during DNA damage, and reaches a nadir during senescence [28]. So we tried to test whether survivin was involved in KLF4-induced cellular senescence. Protein level of survivin (Figure 3A) and mRNA expression (Figure 3B) were both inhibited by KLF4 overexpression. Then, we overexpressed survivin in T-REx-293 KLF4 cells (Figure 3C), and overexpression of survivin could partially recover cell senescence induced by KLF4 (Figure 3D). Additionally, p21 upregulation induced by KLF4 was significantly inhibited (Figure 3E). It has been reported that survivin could inhibit p21 expression at transcription level by directly binding to two p53 binding sites in p21 gene promoter region [29]. In our study, survivin protein could directly bind to the distal and proximal p53 binding sites of p21 promoter in T-REx-293 KLF4 cells, as confirmed by ChIP assay (Figure 3F). T-REx-293 cells were co-transfected with survivin and reporter plasmids (pGL3 p21 5′, pGL3 p21 3′ or pGL3 Basic), and reporter assay showed that the transcription activities of both pGL3 p21 5′, pGL3 p21 3′ were significantly inhibited by survivin (Figure 3G). Our data show that survivin-p21 pathway might contribute to KLF4-induced senescence.

### KLF4 induces senescence though miR-203-survivin-p21 pathway

**Figure 4:**
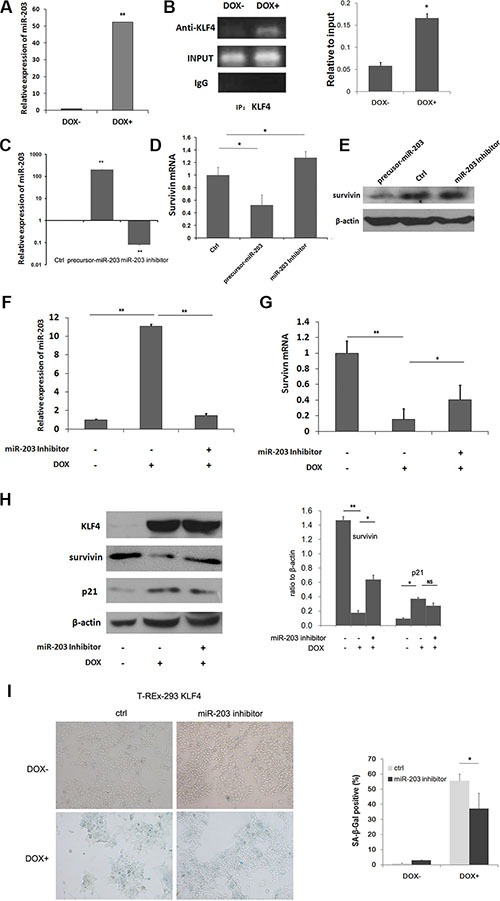
Mir-203 could directly be upregulated by KLF4 and inhibit survivin expression (**A**) Real-time PCR analysis of relative miR-203 expression upon DOX treatment of T-REx-293 KLF4 cells. Bars represent the mean ± SD (*n* = 3). **p < 0.01. (**B**) PCR analysis of direct binding of KLF4 to KLF4 binding sites of miR-203 promoter in T-REx-293 KLF4 cells with/without DOX treatment. Anti-KLF4 antibody or mouse IgG were used for immunoprecipitation. Input DNA was used as positive control, and immunoprecipitation of IgG as negative control. (**C**) Detection of miR-203 in T-REx-293 cells transfected with pre-miR-203 precursor or miR-203 inhibitor by qRT-PCR. Bars represent the mean ± SD (*n* = 3). **p < 0.01. (**D**) Real-time PCR analysis of survivin mRNA regulated by miR-203. Bars represent the mean ± SD (*n* = 3). *p < 0.05,**p < 0.01. (**E**) Western blotting analysis of protein expression of survivin. (**F**) Expression of miR-203.T-REx-293 KLF4 cells were pre-transfected with miR-203 inhibitor or its scrambled control for 24 h, and then treated with DOX for 24 h. Bars represent the mean ± SD (*n* = 3). **p < 0.01. (**G**) mRNA level of survivin detected by qRT-PCR. Bars represent the mean ± SD (*n* = 3). *p < 0.05,**p < 0.01. (**H**) Survivin and p21 protein expression analyzed through Western blotting. (**I**) Representative SA-β-gal staining photos(magnification 100×) and percentage of senescence cells. T-REx-293 KLF4 cells were pre-transfected with miR-203 inhibitor or its scrambled control for 24 h, and then induced by DOX for 72 h. Bars represent the mean ± SD (*n* = 3).

Regulation of miRNA expression has been reported in cell senescence [30, 31]. Here, microRNAs were evaluated with real-time PCR and microRNA array for T-REx-293 KLF4 cells. Upon KLF4 induction, thirty-two miRNAs were upregulated while eight downregulated above five folds according to Applied Biosystems microRNA array data (Table 1). miR-203 has been reported to target survivin [32, 33], and also induce senescence in human melanoma cells [34]. Then we confirmed by real-time PCR that miR-203 was up-regulated about 52 fold after KLF4 induction inT-Rex-293 KLF4 cells (Figure 4A). Moreover we found eight putative binding sites, matching the consensus DNA binding sequence of KLF4, 5′-(G/A)(G/A)GG(C/T)G (C/T)-3′ [35], within 2000bp upstream of miR-203 gene (Figure S3). ChIP assay showed that KLF4 could directly bind to -189bp to +11bp fragment of miR-203 gene (Figure 4B). To test whether miR-203 can target survivin or not, pre-miR-203 precursor and miR-203 inhibitor were transfected into T-REx-293 cells, respectively (Figure 4C). As shown in Figure 4D, mRNA expression of survivin was inhibited by pre-miR-203, while inhibition of miR-203 could increase survivin expression, the similar result was shown in protein level (Figure 4E). To further explore the role of miR-203 in KLF4-induced senescence, miR-203 inhibitor was transfected into T-REx-293 cells for 24h. miR-203 upregulation by KLF4 was inhibited (Figure 4F). The inhibition of survivin was partially recovered in both mRNA and protein level (Figure 4G and 4H). p21 was reduced (Figure 4H), and senescence-associated β-galactosidase activity (SA-β-gal) positive cells decreased in cells pretreated with miR-203 inhibitor (Figure 4I). A similar result was also detected in T-REx-HeLa cell line (Figure S4). Moreover, miR-203 overexpression could induce cellular senescence in AGS and SW480 cells, and reduced survivin mRNA and protein expression were also detected in these cells (Figure S5). Our data demonstrate that miR-203-survivin-p21 pathway may be responsible for KLF4-induced senescence.

**Table 1:** MicroRNAs that changed more than 5 fold upon KLF4 induction of T-REx-293 KLF4 cells

Up-regulated microRNA	Fold change	Up-regulated microRNA	Fold change
hsa-miR-200a	89.6194	hsa-miR-22	9.7733
hsa-miR-429	79.1522	hsa-miR-512-3p	9.7317
hsa-miR-200b	61.6227	hsa-miR-200c	8.631
hsa-miR-200a*	37.9009	hsa-miR-96	8.0666
hsa-miR-409-3p	35.9378	hsa-miR-376a	7.96
hsa-miR-150	34.4571	hsa-miR-888	7.591
hsa-miR-203	29.7177	hsa-miR-642	7.2148
hsa-miR-891a	17.8182	hsa-miR-376c	7.0399
hsa-miR-539	15.504	hsa-miR-200b*	6.6243
hsa-miR-133a	15.3815	hsa-miR-183*	6.3102
hsa-miR-375	14.6615	hsa-miR-508-3p	6.2253
hsa-miR-675	14.421	hsa-miR-149	5.9639
hsa-miR-134	13.1275	hsa-miR-133b	5.6385
hsa-miR-16-1*	13.0065	hsa-miR-935	5.2572
hsa-miR-141	10.5047	hsa-miR-483-5p	5.2109
hsa-miR-372	10.0942	hsa-miR-509-3p	5.1512

Down-regulated microRNA	Fold change	Down-regulated microRNA	Fold change
hsa-miR-107	28.32861	hsa-miR-486-5p	5.777008
hsa-miR-548b-5p	9.124088	hsa-miR-579	5.763689
hsa-miR-32	6.613757	hsa-miR-596	5.184033
hsa-miR-28-5p	6.17284	hsa-miR-374b*	5.08647

### KLF4 expression predicts better prognosis in ESCC & CRC

**Figure 5:**
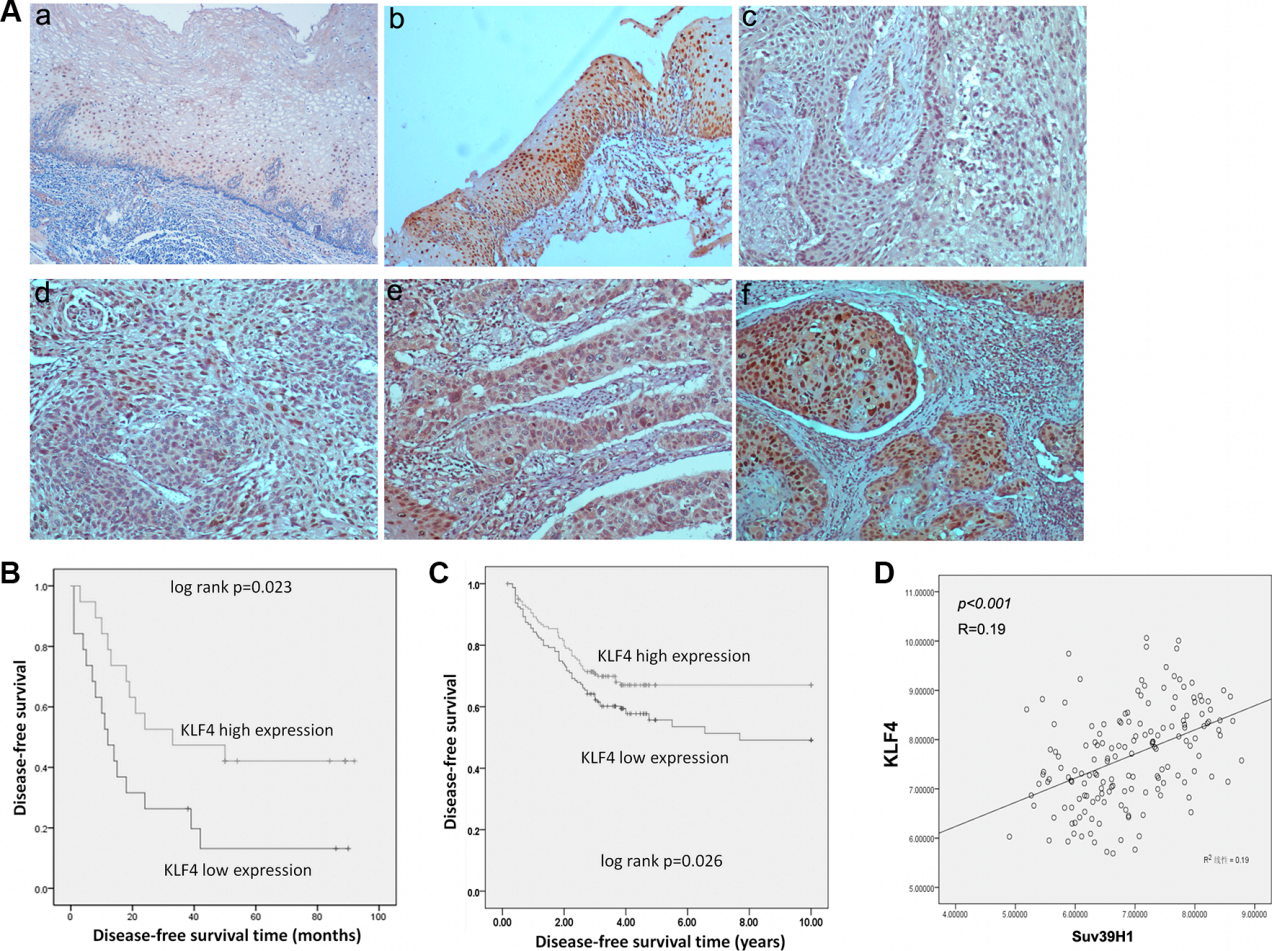
KLF4 expression predicted better prognosis of ESCC and CRC patients (**A**) Representive immunohistochemical staining of KLF4 in normal squamous epithelium and squamous cell carcinoma of esophagus. (magnification 100×) a, b, in normal squamous epithelium, the staining of KLF4 was intensely at cell nucleus and cytosol. c-f: grading of staining of KLF4 as 0,1,2,3. (**B**) Disease-free survival curves of KLF4 in 38 ESCC patients, analyzed by Kaplan-Meier method. (**C**) Disease-free survival curves of KLF4 analyzed in GEO database (GSE24551). (**D**) Correlations between KLF4 and Sur39H1 expression analyzed in GEO database (GSE24551).

To evaluate the potentially clinical indication of KLF4 expression, we detected KLF4 expression in esophagectomy specimens of 38 ESCC samples by immunohistochemistry and analyzed relationship between the expression of KLF4 and survival time of ESCC patients. The staining of KLF4 was intensely at cell nucleus and cytosol in normal squamous epithelium of esophagus (a, b), representative expressions of KLF4 in ESCC were shown in (c–f) (Figure 5A) Then, we analyzed the relation between KLF4 expression and disease-free survival of the 38 patients. Remarkably KLF4 expression level was significantly correlated with the disease-free survival of ESCC patients after surgery following with Kaplan-Meier analysis (*p* = 0.023) (Figure 5B). The median recurrence time was 12 months in low KLF4 expression group and 33 months in high expression group. Furthermore, we found similar connection between KLF4 mRNA expression and disease-free survival time of CRC patients in the openly existing GEO database (GSE24551, http://www.ncbi.nlm.nih.gov/geo/). Of 320 CRC patients, we divided them into two groups based on the median value of KLF4 expression, and found KLF4 expression levels were also significantly correlated with the disease-free survival of the CRC patients (*p* = 0.026) (Figure 5C). Moreover, KLF4 was positively correlated with Suv39H1, a senescence maker (Figure 5D). The result implied that KLF4 overexpression might induce senescence and predicted better prognosis in cancer patients.

## DISCUSSION

KLF4 could induce cell senescence during the process of induced pluripotent stem cells in fibroblast cells [22]. Here by constructing a cell model of doxycycline inducing KLF4 expression in T-REx-293 cells, we tried to investigate how KLF4 induced cell senescence. In our study, overexpression of KLF4 could indeed promote cellular senescence in T-REx-293 and other cell lines. It seems that increased expression of p21 by KLF4 is likely the main pathway for KLF4 induced senescence in this system. However, it was recently reported that KLF4 deficiency caused premature senescence in mouse embryonic fibroblasts by inducing oxidative DNA damage and activating p53/p21 pathway [23]. As an important transcription factor, while overexpression of KLF4 could directly induce p21 and senescence, knockout of KLF4 may also activate upstream of p53 and induce p53/p21 pathway. Except for KLF4, there are also several other genes which could induce senescence whether overexpression or inhibition, such as PTTG [36], and c-Myc [37].

Recently it was reported that KLF4 K409Q together with TRAF7 mutations were harbored in most secretory meningioma patients [39]. This scenario was confirmed by the others [40]. Since residue K409 is located within the first zinc finger and direct DNA binding motif of KLF4, it implies that the K409Q mutation might destroy the binding between KLF4 and DNA. However, according to our results, sole KLF4 409Q mutation had no significant effect on both p21 expression and senescence in HEK293 and HeLa cells. There were several reports about KLF4 mediated the transactivation of p53 on p21 promoter [13, 41]. In our study, KLF4 could induce p21 expression without affecting p53 expression. Even in HeLa cells in which p53 should be functionally suppressed by HPV [42], KLF4 could still induce cellular senescence. Besides, KLF4 could also induce senescence in human colon cancer cell line HCT116 (p53 −/−) (data not shown). But when we knocked down p21, the senescence percentage was significantly reduced. It was reported that p21 loss could even convert KLF4 from cell-cycle inhibitor into oncoprotein [1]. So p21 seems to be the essential target of KLF4 during cellular senescence in our system.

Survivin is one of KLF4′s target genes [27]. Survivin involves in several disparate molecular networks of cellular division, intracellular signaling, and apoptosis [43, 44]. Survivin was reported to be downregulated during drug induced senescence, and to be overexpressed in cells escaping from senescence [28]. It was also reported that senescence was a reversible process controlled by survivin, so that overexpression of survivin in senescent cancer stem cells could promote tumorigenesis [45]. Inhibition of survivin usually correlated with upregulation of p21, especially during senescence [46, 47]. Our results showed that KLF4 inhibited expression of survivin and p21 was upregualted. Moreover, we proposed that direct binding of survivin protein to p21 promoter region might be involved in the senescent process. Although survivin is not a classical transcription factor, it could bind to two p53 binding sites of p21 gene promoter, inhibiting p21 expression [29].

KLF4 has been reported to regulate microRNA expression, such as miR-206, and miR-146a [48, 49]. In our system, we find some microRNAs as potential targets of KLF4. Some of these microRNAs could induce senescence, such as miR-203 [34], miR-141, and miR-22 [50, 51]. Among them, miR-203 can target survivin [32]. In our study, we confirmed miR-203 inhibited survivin expression, and KLF4 could induce miR-203 expression. Combining with our previous data that KLF4 directly inhibited survivin expression [32], our results demonstrate that KLF4 could regulate survivin through direct or indirect manner during inducing cell senescence.

As a tumor suppressor gene, KLF4 was downregulated in cancer tissues compared with noncancerous tissues in gastrointestinal track [52]. Decreased KLF4 expression highly related to a poor survival in gastric cancer and colon cancer [52, 53]. In our study, we found that KLF4 expression significantly related to disease-free survival of ESCC patients, and high expression of KLF4 showed a better disease-free survival. Besides, using GEO databases, we found that KLF4 mRNA expression also correlated with disease-free survival of CRC patients, which further confirmed our hypothesis. Cellular senescence is usually thought to be a barrier of tumor initiation and development. Then it is reasonable to speculate that the senescent cells in cancer tissue may predict a better survival time of cancer patients. Except for KLF4, overexpression of DEC1, p21 and ING1, which could induce cell senescence, are associated with a better survival in certain cancers, indicating that senescence may also have a protective function in cancer development [54–57]. Although more evidence of how KLF4 induce senescence *in vivo* is needed, our results strongly implied that KLF4 might resist cancer progression through promoting cellular senescence.

**Figure 6:**
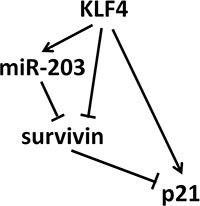
The signaling network of KLF4-inducing senescence KLF4 could induce senescence through a complex regulatory network: direct induction of p21, inhibition of survivin that indirectly induced p21, and induction of miR-203, which inhibited survivin and induced p21 overexpression.

Taken together, our study described a model that KLF4 could induce senescence through a complex regulatory network: direct induction of p21, inhibition of survivin that indirectly induced p21, and induction of miR-203, which targeted survivin and induced p21 expression (Figure 6). Moreover, overexpression of KLF4 is significantly consistent with better prognosis in ESCC and CRC patients. Our data shed light on the function of KLF4 of inducing cellular senescence *in vitro* and preventing tumor progression *in vivo.*

## MATERIALS AND METHODS

### Plasmids, pre-miR precursors and antisense oligonucleotides

pcDNA4 HA-KLF4 was generated from double digestion of pcDNA3 HA-KLF4 [27], followed by ligation to pcDNA4™/TO/myc-His B (Invitrogen, Carlsbad, CA). pcDNA4 HA-KLF4-K409Q plamids was further constructed containing 1225 A to C mutation. Survivin expression plasmid pcDNA3.0 survivin, pGL3 p21 5′, pGL3 p21 3′ and p21 shRNA plasmids pSilencer3.0 shp21 were constructed as described previously [58, 59]. The pre-miR-203 precursor, pre-miR negative control, miR-203 inhibitor and scrambled oligonucleotides of miR-203 were purchased from Ambion (Austin, TX, USA).

### Cell lines and transfection

T-REx™-293 and T-REx™- HeLa were cultured in DMEM (Gibco). All medium contained 10% fetal bovine serum and supplemented with 100 U/mL penicillin and 100 μg/mL streptomycin. Cells were maintained at 37°C in a humidified incubator with 5% CO. Lipofectamine2000 (Invitrogen) was used for plasmids and oligonucleotides transfection; siPORT^™^ NeoFX^™^ transfection agent (Ambion) was used for pre-miR-203 precursor, miR-203 inhibitor, scrambled oligonucleotides (Ambion) transfection, according to the manufacturer's instructions.

For the establishment of stable transfected cell lines, cells were selected with Zeocin (100 μg/ml, Invitrogen) after transfection. 2 μg/ml doxycycline (Sigma Aldrich) was used to induce KLF4 over-expression. The resistant cell lines were designated as T-REx-293 KLF4, T-REx-293 PC4, T-REx-HeLa KLF4 and T-REx-HeLa PC4, respectively.

### Protein preparation and Western blotting

Cells were harvested and lysed in RIPA buffer (Sigma Aldrich) and protein concentrations were measured by a BCA protein detection kit (Pierce, Rockford, IL, USA). Proteins were separated by SDS-PAGE and transferred to nitrocellulose membranes. Primary antibodies against KLF4 (1:1000, Santa Cruz), β-actin (1:5000, Sigma Aldrich), p21 (1:1000, Cell Signaling), HA-Tag (1:1000, Santa Cruz), Cyclin E1(1:1000, Abcam), p53 (1:1000, DAKO), p16 (1:1000, Santa Cruz), Suv39H1 (1:1000, Santa Cruz), survivin (1:1000, Abcam) and horseradish peroxidase conjugated secondary antibodies (Zhongshan, Beijing, China) were used to detect specific proteins according to the standard procedures. Finally, the membranes developed with a Luminol Detection System (Santa Cruz). Protein expression was quantified using a Gel EDAS 290 analysis system (Cold Spring Harbor Laboratory, Cold Spring Harbor, NY, USA) and Gel-Pro Analyzer 3.1 software (Media Cybernetics, Silver Spring, MD, USA).

### Senescence-associated β-galactosidase (SA-β-Gal) staining assay

Cultured cells were seeded in 6-well plates and either transfected with intented plasmids, pre-miR precursors, antisense oligonucleotides, or treated with 2 μg/ml doxycycline. After 72 h, the cells were stained with Senescence Cells Histochemical Staining Kit (Sigma) according to the manufacturer's procedure.

### Cell growth and MTT assay

T-REx-293 KLF4, T-REx-293 PC4 cells were plated into 24-well plates at a density of 50 cells/mm^2^ in triplicate with or without 2 μg/ml doxycycline. Cells were harvested every day and cell numbers were counted. For MTT assay, 2,000 T-REx-293 KLF4, T-REx-293 PC4 cells were seeded into 96-well plates and with or without 2 μg/ml doxycycline treatment for 1 to 6 days. Then, 20 μl 3-(4,5-dimethylthiazol-2-yl)-2,5-diphenyltetrazolium bromide (MTT) (5 mg/mL) was added to each test well, and incubated for 4 h at 37°C. The supernatant was discarded and 150 μl dimethyl sulfoxide (DMSO) was added to each well to solubilize the crystals for 10 min at room temperature and measured at a wavelength of 570 nm with the model 680 microplate reader (Bio-Rad, Hercules, CA, USA).

### Colony formation assay

T-REx-293 KLF4 cells were seeded into 10 cm cell culture dishes of 2000 cells/well in triplicate with or without 2 μg/ml doxycycline. After about two weeks, obvious clones in control wells could be observed, and then cells were washed with PBS and fixed with formaldehyde. Then the cells were stained with Giemsa staining solution and clones were counted.

### BrdU incorporation assay

BrdU Cell Proliferation Assay (Calbiochem, San Diego, CA, USA) kit was used to detect newly synthesized DNA *in vitro*, T-REx-293 KLF4 cells in a 96 well cell culture plate (5,000 cells per well) were treated with 2 μg/ml doxycycline for 0 to 5 days, and then incubated with medium containing BruU for 6 h and processed according to the instructions of the manufacturer. The signal was detected using a model 680 microplate reader (Bio-Rad) at dual wavelengths of 450/570 nm.

### Total RNA preparation

Total RNA was prepared using Trizol Reagent (Ambion) in accordance with the manufacturer's protocol. Concentration was measured by NanoDrop 2000 (NanoDrop, Wilmington, DE, USA).

### TaqMan Real-time PCR microRNA array

T-REx-293 KLF4 cells with or without 2 μg/ml doxycycline treatment for three days were harvested and washed in cold sterile phosphate buffered saline (PBS), and then miRNA was isolated using a mirVana RNA isolation kit (Ambion). The stem-loop RT-PCR based TaqMan MicroRNA Arrays (Applied Biosystems, Foster City, CA, USA) representing the 663 mature miRNAs in a two-card set of arrays (Array A and B) were used. RT-PCR reactions were performed according to the manufacturer's instructions. All reagents were obtained from Applied Biosystems. The quantitative miRNA expression data were acquired using the ABI 7900HT SDS software (Applied Biosystems). The Ct value of an endogenous control gene (Mamm U6) was subtracted from the corresponding Ct value of the target gene resulting in the Ct value which was used for relative quantification of miRNA expression. The clustering analysis was performed using a hierarchical method and average linkage [60]. The fold changes in gene expression were calculated using the 2^−ΔΔct^ method [61].

### Real-time PCR

Total RNA was prepared using Trizol Reagent (Ambion) in accordance with the manufacturer's protocol. Concentration and purity of total RNA samples were measured by NanoDrop 2000 (NanoDrop). Total RNA was reverse-transcribed to cDNA with M-MLV reverse transcriptase (Promega, Fitchburg, WI, USA), according to the manufacturer's instructions. Power SYBR Green Master Mix (Applied Biosystems) was used for real-time PCR applications. β-actin was used to normalize data. Sequences of the PCR primers were as follows: p21 forward: 5′- GGCTCCTTCCCATCGCTGTCA -3′, and reverse: 5′-GTCACCCTGCCCAACCTTAGA-3′; survivin forward: 5′-AGGACCACCGCATCTCTACAT-3′, and reverse: 5′-AAGTCTGGCTCGTTCTCAGTG-3′; β-actin forward: 5′-GGCGGCACCACCATGTACCCT-3′, and reverse: 5′-AGGGGCCGGACTCGTCATACT-3′.

For miRNA analysis, reverse transcription and stem-loop real-time RT-PCR were performed as described [62] using TaqMan MicroRNA individual assays, TaqMan MicroRNA Reverse Transcription kit, and TaqMan Universal PCR master mix without AmpErase UNG (Applied Biosystems). For normalization, U6 small nuclear RNA was used as control. The quantitative real-time PCR of each sample was performed in triplicate on an Step One Plus Real-Time PCR Systems (Applied Biosystems) according to the manufacturer's protocol.

### Chromatin immunoprecipitation (ChIP) assay

ChIP assay was conducted with the ChIP-IT^™^ Express kit (Active Motif, Carlsbad, CA, USA) according to the manufacturer's protocol. The antibodies for immunoprecipitation include an anti-KLF4 (Santa Cruz), anti-survivin (Abcam) antibodies. PCR was performed with primers amplifying intended promoter regions. Primers specific for putative KLF4 binding sites and survivin binding sites upstream of p21 promoter were reported previously [29, 63].

PCR primers used for KLF4 binding sites to microRNA-203 promoter were: forward: 5′-ATCCCCC AGCGCCAGGCGAG-3′, and reverse: 5′-GTCCCCAA CACCGTCGGTTC-3′. ChIP experiments were repeated at least three times and representative results were shown.

### Tissue sample collection and immunohistochemistry

Esophageal squamous cell carcinoma (ESCC) tissues from 38 patients who had received esophagectomy were collected in Cancer Hospital, Chinese Academy of Medical Science from December, 2000 to June, 2001. The follow-up period was from 3 months to 92 months, with a mean period of 39.6 months. The disease-free survival time was defined as the time between diagnosis of esophageal squamous cell carcinoma and recurrence of disease. The study was approved by the medical ethics committee of Cancer Hospital, Chinese Academy of Medical Science.

Slides were probed with anti-KLF4 antibody (1:100, Santa Cruz) and assessed by two individual investigators. KLF4 immunoreactivity was scored 0~3 by the color and percentage of cells showing distinct nuclear and/or diffuse cytoplasmic immunohistochemical reaction. Scores of 0~1 were defined as “low expression”, and scores of 2~3 as “high expression”.

### Statistical Analysis

Statistical analysis was performed using SPSS 18.0 software (SPSS, Chicago, IL, USA). Data were presented as mean ± s.d of at least three independent experiments. Differences between the two groups were examined by Student's two-tailed non-paired *t*-test and a nonparametric test (Mann-Whitney *U*-test for two groups). Survival analysis was carried out using Kaplan-Meier method with log-rank test. A *p* value < 0.05 was considered statistically significant.

## SUPPLEMENTARY MATERIALS FIGURES


