# Supplementary material for: Overexpression of KLF4 promotes cell senescence through microRNA-203-survivin-p21 pathway
Source: Oncotarget. 2016 Aug 11;7(37):60290–302. doi: 10.18632/oncotarget.11200 (PMC5312384; doi:10.18632/oncotarget.11200)
Supplement: Supplementary file 1 [file oncotarget-07-60290-s001.pdf]

Overexpression of KLF4 promotes cell senescence through microRNA-203-survivin-p21 pathway

Supplementary Materials

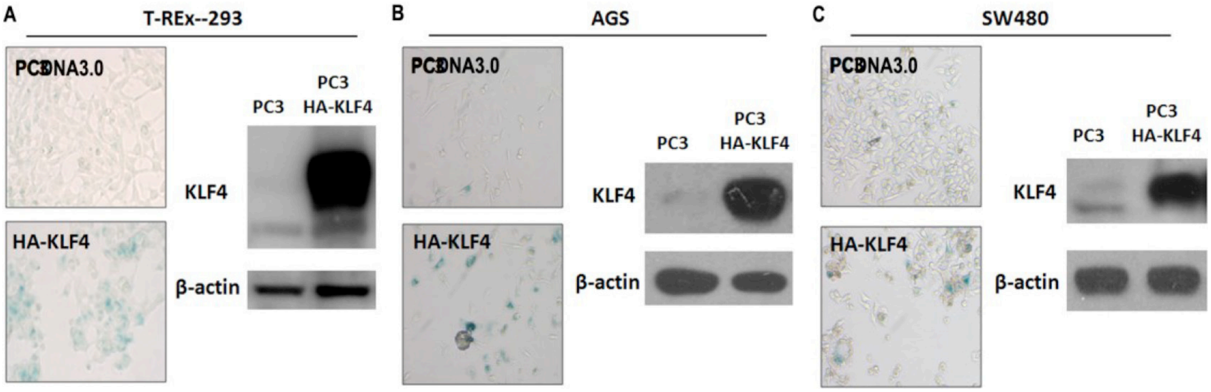

Supplementary Figure S1: Transient overexpression of KLF4 induced senescence. (**A**) T-REx-293, (**B**) AGS, and (**C**) SW480 cells were plated into 6-well plates, and transfected with pcDNA3 HA-KLF4 and pcDNA3 plasmids. Three days after transfection, cellular senescence was detected by SA-β-Gal staining assay (magnification 100×), while KLF4 expression was detected by Western blotting.

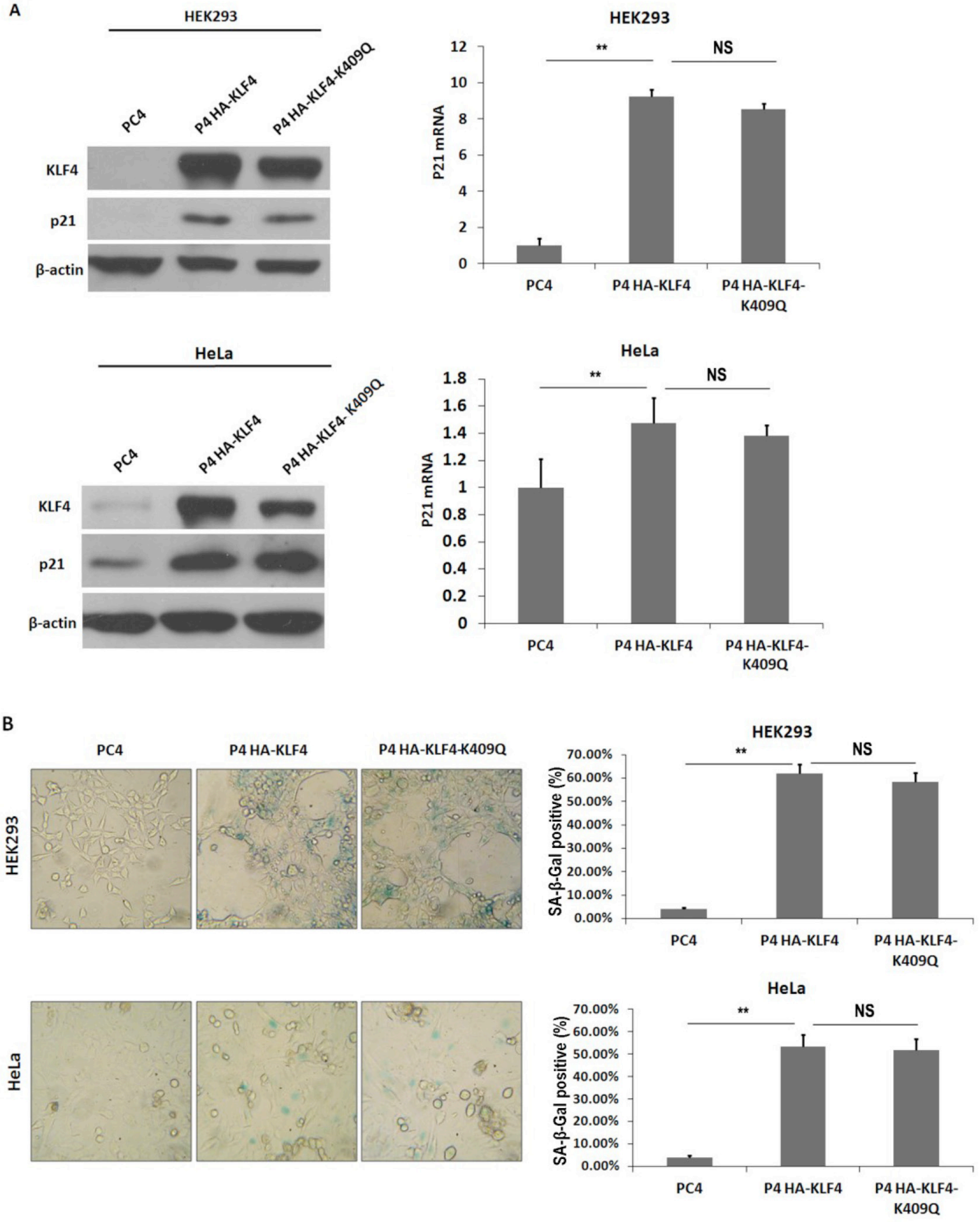

Supplementary Figure S2: KLF4 K409Q mutation did not affect KLF4-induced senescence in HEK293 and HeLa cells. (**A**) Expression of p21 were detected. HEK293 and HeLa cells were transient transfected with pcDNA4, pcDNA4 HA-KLF4, or pcDNA4 HA-KLF4 K409Q plasmid respectively, cells were harvested after 72 h. Bars represent the mean ± SD ($n = 3$). $^{**}p < 0.01$. (**B**) Cellular senescence of HEK293 and HeLa cells detected by SA-β-Gal staining assay and percentage of senescence cells. Bars represent the mean ± SD ($n = 3$). $^{**}p < 0.01$.

```
-2000  GGACTGGGCTAGACCAGAACTTAGAATTAAGGAGTGGTGGCCAGGCTTGGTGGCTCACAC
-1940  CTGCAATCCCAGCACTTTGGGAGGCCGAGGCGGGTGGATAGCTTGAGCCTAGGAGTTTGA
-1880  GAACAGCCAACATGGCGAAACTCTGTCTCTTAAAAAATAAATAAAAACATTAGCCAGGTG
-1820  TGGTGGTTTGTGTCTGTGGTCCCAGCTACTTGGGAGGCTGAGGTGGGAGAATCGCTTGAA
-1760  CTGGGAAGATGGAGGTTGCAGCGAGCCAAGATCGCACCACTGCACTCCAGCCTGGGCAAC
-1700  ACAGCAAGACTAGGTCTCAAAAAAAAAGAAAAAAAAAAAGAAAAGAAGAGAGGTGGGTTC
-1640  TTCTTGCCCATGCAGTTGTGTTGGGGGAGGAGCTCCCAAGCTCCTGTTCTTGATCTCAAG
-1580  CTCCCCTATGCCCACTTCCATCCCCACCCCCATGCCGCAGCCCCTCGGACTCCACACCCA
-1520  GGCACTGAGACCCACAGATGCAGCGATGGCAAGGCCTGTCATCCCTTCTCTACCACCAAG
-1460  AGCTGTGCAATCCCCAGAAGTCACTGTCCCTCCCTGAGCCTCAGTTTCCCCAACTGCAAA
-1400  ATGAAAATAACAAAACCTTTCTGATTCCCTTTCCAGGGTGGCTGCGGTGGTTGAGCCACA
-1340  CGCGGCCCATGTGGAAATGTCTGGGGGGTCCCTCAGATCTGGTGGCTGTGTTCTGGTCTG
-1280  GGGCTCACCTTGGCTCCTGAGCACTTGGCTCCTGGCTGGCTGGGCGGGTGGTGGGATGGC
-1220  GTGTCACCGCCCTGCACTGGAATGAATGGTCACCACCCTGCTGCCCAACCCCATACAGAC
-1160  ACACTAATGGCTCCAGACTTGGGGCAAGTGCTGAAGGGGGCTCTAGGGTATTTCCTAGAG
                                                            1
-1100  CCTGAGCCCTCCCCTCAGCTGCCATGTTCTGCTGAAGCAGAGTCCCTGGGAGGCGCCACT
-1040  GGTCAGGCCTGGACACCTCCAGGTGACTAAGTGGGTAGGACCGGCAGGGCCAGCTGCGCC
-980   AGGACGTTCGGGGCTGGGCTGTGAGTGGGGGGAGCGAGGCTCAGGCCCTTGCTGCGTGAG
                  2
-920   GGGGTAGGGCGTCCCTCCCCCCGCGTGGGAAATGAGGAGGCAGGTACGGGGCTACCCCTG
-860   TGGGCTGCGGGGTGGGGGTTCAGGGTCTGAGAAGCAGAAACCTCCCTCCCTCCAGTTTCA
-800   CTTTCTCTTTACCCCTCCCCTTCCCCCTCCCACGAAGTTCCCTTTGAAGTGAGAGGGGCT
-740   GGGGTGGGTGTGTCCAGCCCAGCCCCCACACCCACCGGAGAGCTAGCGCGGCCCTGGGCT
-680   CCTGTCGGGCGCTGGTCCTCACCTGTTCCGGCCACCCTGGGCCTCGACCCGGCCAAGGTG
-620   GAGCCCCGGGCCCTCCGTGCGCCCGAGCACCCCCGGCCCAGACGAGACGGTTCGGGCGTG
-560   GCCCGGCGGGGACCCAGAACCCGGGAGGCCAGGTGCGCCCAGGCCAGGCGCTGGAGGCTG
         3
-500   GGGCGCCGGATGGGGCGGGGCGGCCGCTGAGTCAGGGCCACCCCCGCGCGGGCGCGCGGA
-440   GCAGGTCCCCGGGCCGTGGAGGATCAGTCGCGGGACCTATGGGCCCGGGAGCCGCCCCGC
-380   CCGGAAAAACCGGTCGGGCCAGCGCGGCGCGGGCACATTCCGGCGCCGGGAAGGGCGGCT
                  4
-320   GCGCCGCATAAAAGGCGCCGCCGCGCTGGCCACCGCCGCTCGGAGCTCAGCCGCCCTGCC
-260   ACGCGGGGCCCGCGGGACGAGGGGACGCCGGGGCCAGCGAGGACGCGGCGGGGCTGGGCT
                                    5       6
-200   TGGCGGCTGGGATCCCCCAGCGCCAGGCGAGGGCGTCTAAGGCGTCCGGTACGGCGTCGG
                                  7
-140   GGCCGGGTGGCTGCAGCAGGGCAGGGGGTGCGCGACCAGCGGGGATCTGGGCGCAGGGGC
                                                8
-80    CGGTCCCCGGGATCCGCAGGCGACGCGGGCGGTCCCAAGGGCGTCGGGGGCTCCTCTCTC
-20    CGCAGCTCGGCGAACCGACGGTGTTGGGGACTCGCGCGCTGGGTCCAGTGGTTCTTAACA
+40    GTTCAACAGTTCTGTAGCGCAATTGTGAAATGTTTAGGACCACTAGACCCGGCGGGCGCG
+100   GCGACAGCGA
```

Supplementary Figure S3: Promoter sequence analysis of human miR-203 gene. DNA sequence of 2000 bps upstream of the human miR-203 gene were analyzed and eight potential KLF4 binding sites was noted.

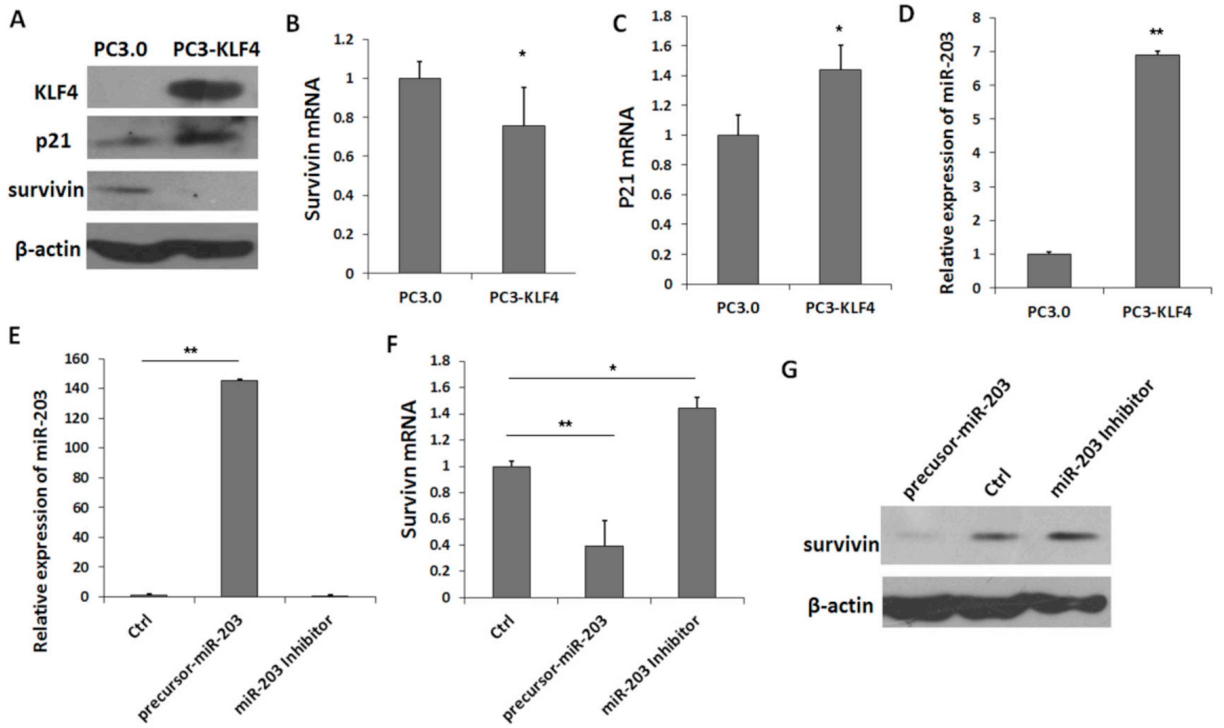

Supplementary Figure S4: MiR-203-survivin-p21 pathway was regulated by KLF4 in T-REx-HeLa cells. T-REx-HeLa cells were transfected with pcDNA3 HA-KLF4, and empty pcDNA3.0 plasmids. After 72 h, (**A**) protein level and mRNA level of (**B**) survivin and (**C**) p21 mRNA level was detected, and (**D**) miR-203 expression level was also detected by qRT-PCR. (**E**) T-REx-HeLa cells were transfected with pre-miR-203 precursor, its scrambled control, and inhibitor. After 48 h, relative miR-203 expression level was detected. (**F**) T-REx-HeLa cells were transfected with pre-miR-203 precursor, scrambled control, and inhibitor. After 48 h, relative survivin expression at mRNA level and (**G**) protein level was detected. Bars represent the mean ± SD ($n = 3$). *$p < 0.05$,**$p < 0.01$.

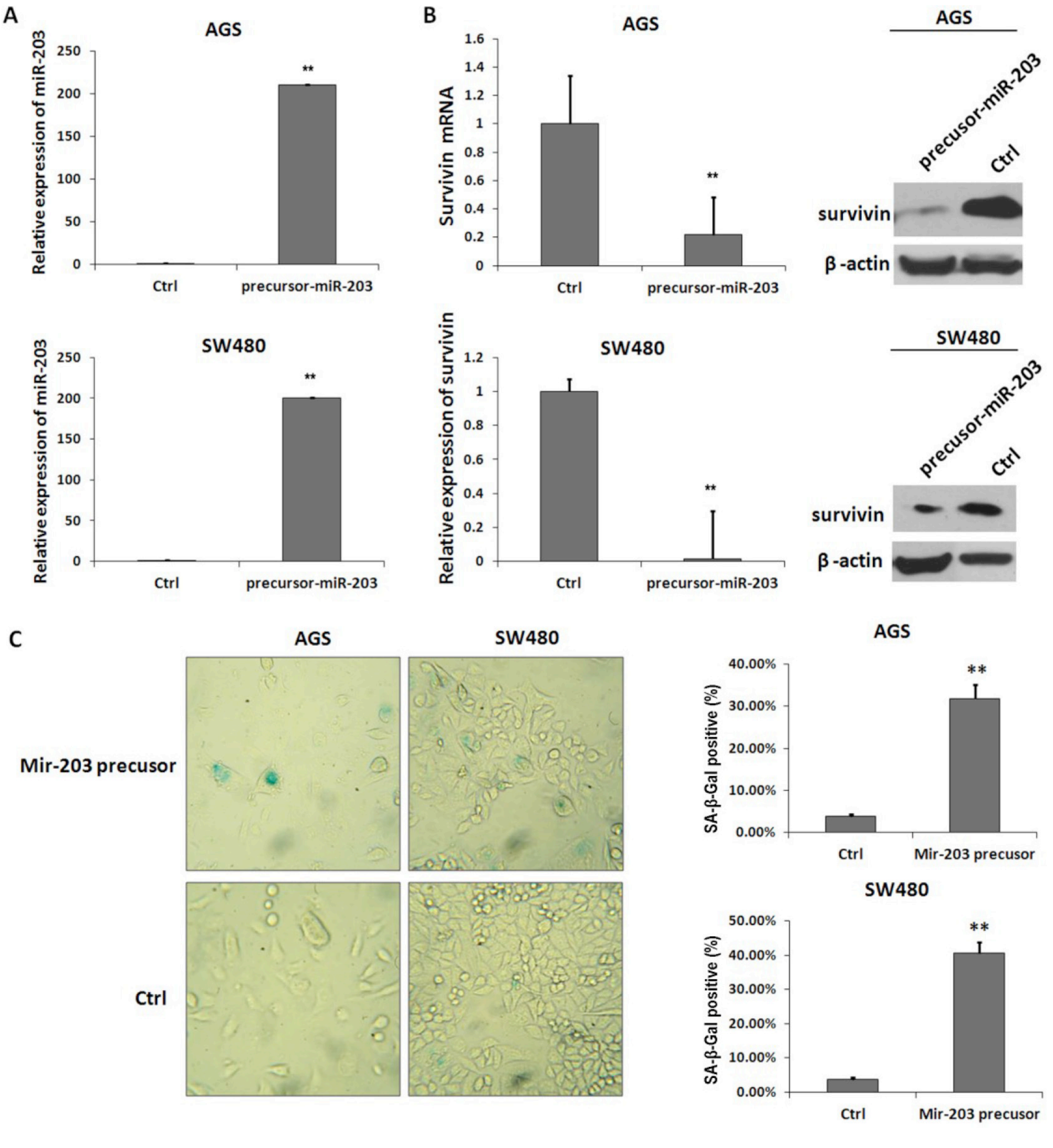

Supplementary Figure S5: Overexpression of miR-203 inhibited survivin expression and induced senescence in AGS and SW480 cells. (**A**) Detection of miR-203 expression. AGS and SW480 cells were transfected with miR-203 pre-miR-203 precursor and its scrambled control and harvested for Real-time PCR after 72 h. (**B**) Survivin mRNA level and protein regulated by miR-203. (**C**) SA-β-Gal staining and percentage of senescence cells. All experiments were performed in triplicated. ***p* < 0.01.
